# “A Life More Ordinary” Processes of 5-Year Recovery From Substance Abuse. Experiences of 30 Recovered Service Users

**DOI:** 10.3389/fpsyt.2019.00689

**Published:** 2019-09-18

**Authors:** Jone Bjornestad, Thomas Solgaard Svendsen, Tale Ekeroth Slyngstad, Aleksander H. Erga, James R. McKay, Sverre Nesvåg, Alexander Waagan Skaalevik, Marius Veseth, Christian Moltu

**Affiliations:** ^1^Department of Social Studies, Faculty of Social Sciences, University of Stavanger, Stavanger, Norway; ^2^Centre for Alcohol and Drug Research, Stavanger University Hospital, Stavanger, Norway; ^3^The Norwegian Centre for Movement Disorders, Stavanger University Hospital, Stavanger, Norway; ^4^Department of Psychiatry, Perelman School of Medicine, University of Pennsylvania, Philadelphia, PA, United States; ^5^Philadelphia VA Medical Center, Philadelphia, PA, United States; ^6^Department of Clinical Psychology, University of Bergen, Bergen, Norway; ^7^Department of Psychiatry, District General Hospital of Førde, Førde, Norway

**Keywords:** substance use, substance use disorder, drug reduction, drug change, recovery, long-term recovery, functional factors, social factors

## Abstract

**Background:** Studies investigating the subjective experiences of long-term recovery from substance use disorder are scarce. Particularly, functional and social factors have received little attention.

**Objectives:** To investigate what long-term recovered service users found to build recovery from substance use disorder.

**Material and Methods:** The study was designed as a phenomenological investigation subjected to thematic analysis. We interviewed 30 long-term recovered adult service users.

**Results:** Our thematic analysis resulted in five themes and several subthemes: 1) paranoia, ambivalence and drug cravings: extreme barriers to ending use; 2) submitting to treatment: a struggle to balance rigid treatment structures with a need for autonomy; 3) surrendering to trust and love: building a whole person; 4) a life more ordinary: surrendering to mainstream social responsibilities; and 5) taking on personal responsibility and gaining autonomy: *it has to be me, it cannot be you*.

**Conclusions:** Our study sample described long-term recovery as a developmental process from dependency and reactivity to personal autonomy and self-agency. The flux of surrendering to and differentiating from authority appeared to be a driving force in recovery progression. Participants called for treatment to focus on early social readjustment.

## Introduction

Knowledge of long-term recovery after substance use disorder (SUD) is essential to personalized care. Recovery from SUD can be understood as a person’s active processes in managing the disorder and its residual effects. Ideally, such processes lead to perceived empowerment and contributory citizenship (1, 2). Empirical evidence, however, indicates recovery as non-linear and cumbersome: the threat of relapse continuously looms, and contributory citizenship is less common (3, 4). Factors associated with recovery are supportive social networks, safe residence (5), activities that build abstinence and self-esteem, as well as improved coping strategies. In order for interventions to be efficient, these need to be performed within a framework of tailored and well-timed care (4, 6–9).

Current evidence on recovery from SUD is mainly derived from short-term correlation studies of factors associated with reduced substance use or leading to abstinence (10–18). Controlled, longitudinal studies including functional and social factors are rare. Few studies have investigated service user experiences of social recovery over time. Given the wide acceptance of functional and social factors as key to effective treatment and citizenship (9,), this forms a knowledge gap in the recovery literature.

This exploratory study is part of an ongoing prospective clinical cohort study investigating long-term course and outcome in a representative sample of individuals with SUD (19, 20). Our informants form a sub-sample of 30 individuals who meet strict criteria of long-term substance abstinence and social recovery. The main aim was to investigate any processes perceived by informants to build long-term recovery. See also Ref. (21), for another article from this study focusing on the participants’ experiences of their close relationships.

## Material and Methods

We used a thematic analytic approach (22, 23) within an interpretative-phenomenological framework (24, 25). The interpretative approach meant that study data were generated both from a reflexive dialog between participants and researchers as well as from a member checking procedure throughout the interview. The phenomenological element entails the collection of significant knowledge from individuals with lived experience of SUD in order to discover and interpret the meaning of such experiences within their broader contexts (26). We developed objectives and procedures within a user-involved research framework (27, 28). We recruited two service users with firsthand knowledge of long-term recovery from SUD (TES and AS). They contributed in developing the interview guide, during the interview process, in analysis, and in finalizing the study. This study was reviewed and approved by the Regional Ethical Committee (2011/1877-REK Vest) and conducted according to its guidelines and those of the Helsinki Declaration (1975). Participants gave their informed written consent.

### Sample and Recruitment

The sample was recruited from the ongoing STAYER study (n = 202), a prospective, naturalistic follow-along SUD study of change trajectories in Rogaland, Norway. Service users were included between March 2012 and December 2015, recruited from outpatient and residential treatment facilities at the start of treatment. Inclusion criteria included: person starting a new treatment sequence, fulfilled criteria for a substance-related use disorder, and age ≥16. Further details of STAYER are published elsewhere (20, 29).

We recruited sub-study participants consecutively at their 4- or 5-year follow-ups. The STAYER team conducted a screening process based on objective criteria for stable substance abstinence and social recovery. Thirty-four eligible candidates were contacted; of these, four individuals refused participation. Sample size was decided on the basis of stability of findings (30), reviewed after 19 and 26 participants. We stopped recruiting after 30 participants because we considered the last four interviews not to contribute substantially new information. In the *Results* section, we refer to 20–30 participants as “most,” 10–19 as “many,” and to 5–9 as “some” participants (31).

### Measures

We used consumption items from the Drug Use Disorders Identification Test (DUDIT-C) (32) to assess drug use, the Alcohol Use Disorders Identification Test (AUDIT-C) (33) for alcohol consumption, the Symptoms Checklist-90 Revised (SCL-90-R) (34) for psychological functioning (Global Severity Index (GSI) reported), the Behavioral Rating Inventory of Executive Function—Adult Version (BRIEF-A) (35) for executive functioning [Global Executive Composite (GEC) reported], and the Satisfaction With Life Scale (SWL) (36) for quality of life (sum score reported).

Drug abstinence was operationalized as DUDIT-C score equal to 0 and AUDIT-C scores ≤2. Relapse was defined as above cutoff scores for either alcohol or drug use during the past 2 years.

Social functioning was operationalized using four variables related to social functioning status: housing, income, friend without addiction, and work/school. Patients scoring “yes” on all four social variables were categorized as adequately socially functioning. Long-term recovery was coded as a single variable of “yes” for all individuals who met both criteria for stable substance abstinence *and* adequate social functioning for the past 2 years.

### Interviews

Interviews were conducted between October 2017 and April 2018. Authors developed a semi-structured interview guide in line with the Miles et al. (37) recommendation, based on existing literature on factors facilitating SUD recovery [e.g., Refs. (6–9)]. The following focus areas guided the interview: (1) person-specific factors; (2) environmental factors; and (3) treatment-related factors. Each theme was introduced with an open-ended question, e.g., “How would you describe the treatment you received?” We used follow-up questions as required, encouraging participants to relate their experiences to relevant contexts, e.g., asking, “Could you tell me more about the link between feeling safe and drug abstinence?” To capture topics not adequately covered by the interview, participants were invited at the end of each session to provide any relevant information that had not yet been elicited. Pilot interviews were conducted with two clinically recovered service users. All interviews were conducted by TES and AS, who received interview training. Interviews (*mean duration*: 57 min; *range*: 27–96 min) were conducted at Stavanger University Hospital (n = 25), at the participant’s home (n = 1), and by telephone (n = 4). Interviews were audio-recorded and transcribed verbatim for the purposes of analysis.

### Analysis

For semantic analysis, we employed a seven-step meaning condensation procedure (23), outlined in **Table 1**. To strengthen the credibility of the study, four of the researchers conducted the analytic procedure independently. During collaborative meetings, the same researchers compared their interpretations, agreed on themes with accompanying quotes, and validated the findings by consensus decision (31), dedicating special attention to steps 4 to 6 presented in **Table 1**. To overcome possible disagreement in the analytic process, we agreed on the following decision rules in the preparatory phases of the study: 1) to resolve minor disagreement by the principle of parsimoniousness and 2) to resolve major disagreement by i) an inductive principle using the raw data as a compass, aiming to select the descriptions most closely reflecting the experience of the phenomena at issue, and ii) further applying the principle of best argument as described above.

**Table 1:** Steps of text condensation.

1.	Becoming familiar with the data through careful reading of the transcribed interviews, forming a main impression of the experiences of the participants, and identification of potential important themes. A theme was defined as a verbalization capturing an important element of the data in relation to the research question, representing a patterned response in the data set.
2.	Generating initial codes, which were defined as the most basic segments of the raw data that could be assessed in a meaningful way regarding the phenomenon.
3.	Searching for and developing candidate themes and subthemes. Remaining codes were set aside at this phase in a separate category for the purpose of being further analyzed and incorporated when appropriate.
4.	Reviewing themes to develop a coherent thematic map and considering the validity of individual themes in relation to the data set.
5.	Defining and naming themes: further refining and defining themes, identifying the essence of themes, identifying subthemes, and summarizing the contents of the main themes into what each researcher considered to best represent participants’ experiences. When our refinements no longer added substantially to the themes, the analytic process was closed.
6.	To determine the relevance of a particular theme, we both counted the frequency of the relevant meaning units combined with our interpretation of how central the theme was perceived to the recovery process.
7.	Last, the tentative model of findings, with illustrative quotes, was sent to two fully recovered service users who served as critical auditors assessing the interpretations made through our descriptions of the central organizing concepts.

TES and AS were selected as critical auditors to review and provide detailed feedback during the analysis and writing process. In accordance with Hill (38), the critical auditors’ role is to ensure the structural validity of findings and that themes successfully represent any important material. Both auditors received basic textual analysis training and participated in several collaborative analysis meetings.

## Results

Demographic, clinical, treatment, psychological, and social variables are displayed in **Table 2**. Reflecting the inclusion criteria of long-term recovery, participants generally showed a positive trend in scores across follow-up.

**Table 2:** Baseline and follow-up demographic, clinical, treatment-related, psychological, and social variables.

		Baseline (N = 30)	Year 1 (N = 30)	Year 2 (N = 30)	Year 3 (N = 30)	End point assessment	
					Year 4 (N = 10)	Year 5 (N = 20)
*Demographics*							
Age		25.9 (5.5)	–	–	–	–	–
Male/female, n		17/13	–	–	–	–	–
Education, years		12.8 (1.8)	–	–	–	–	–
*Substance use history*							
Age of initial use		13.1 (1.8)	–	–	–	–	–
Years of drug use		12.9 (6.0)	–	–	–	–	–
AUDIT score		11.9 (11.4)	3.4 (7.6)	2.3 (4.1)	2.9 (6.8)	4.4 (7.0)	2.2 (3.2)
DUDIT score		29.0 (15.9)	6.6 (13.1)	3.1 (11.5)	1.9 (8.5)	0 (-)	0 (-)
*Treatment*							
Previous treatment attempts		1.3 (2.0)	–	–	–	–	–
Currently outpatient, n (%)		13 (43.3)	17 (56.7)	8 (26.7)	5 (16.7)	2 (20.0)	2 (9.5)
Currently inpatient, n (%)		17 (56.7)	5 (16.7)	4 (13.3)	2 (6.7)	0 (0)	0 (0)
Currently in self-help group^a^, n (%)		13 (43.3)	13 (43.4)	15 (50.0)	10 (33.3)	4 (40.0)	3 (14.3)
*Social variables*^b^							
Permanent housing, n (%)		15 (50.0)	25 (83.3)	25 (83.3)	26 (86.6)	10 (100)	21 (100)
Stable income, n (%)		16 (53.3)	21 (70.0)	27 (90.0)	27 (90.0)	10 (100)	21 (100)
Employed/student, n (%)		5 (16.7)	7 (23.3)	14 (46.7)	19 (63.3)	10 (100)	21 (100)
Abstinent friends^c^, n (%)		24 (80.0)	25 (83.3)	26 (86.7)	27 (90.0)	10 (100)	21 (100)
*Psychological measures*							
SCL90-R GSI		1.2 (0.7)	0.7 (0.7)	0.6 (0.5)	0.5 (0.4)	0.5 (0.4)	0.4 (0.5)
BRIEF-A GEC		67.2 (11.3)	57.2 (11.3)	54.9 (12.6)	51. (10.9)	52.5 (10.5)	50.4 (11.2)
SWLS, sum score		17.5 (6.8)	24.8 (6.7)	24.8 (5.2)	25.2 (5.4)	25.3 (2.7)	27.4 (5.0)

All numbers are mean (SD), unless otherwise specified. SCL-90-R GSI, Symptom Checklist-90 Revised Global Severity Index T-score; BRIEF-A GEC, Behavioral Rating Inventory of Executive Function—Adult Version Global Executive Composite T-score; SWLS, Satisfaction With Life Scale; AUDIT, Alcohol Use Disorders Identification Test; DUDIT, Drug Use Disorders Identification Test.

a Currently in self-help group, such as Narcotics Anonymous (NA)/Alcoholics Anonymous (AA) and the like.

b Social variables are positive responses to yes/no questions.

c Friends without a history of substance use.

### Thematic Analysis

Long-term recovery was described as starting with detoxification and moving toward perceived citizenship. A recurring, dynamic process of surrender and differentiation seemed key to participants achieving this transition. Here, surrender refers to accepting and complying with certain structures of authority, community, social network, care, or belief systems, which were at some point perceived to facilitate recovery. Differentiation refers to a desire to disentangle from the very same structures, usually as a result of subjectively perceived progress. Dynamics of surrender and differentiation constitute the most abstract thematic level describing positive change in the result section. This overarching theme comprises five subthemes.

### Paranoia, Ambivalence, and Drug Cravings: Extreme Barriers to Ending Use

All participants experienced a period of intense biological abstinence symptoms when coming off drugs. They described this as a state of physical terror and mental chaos, with intense negative emotions including paranoia, extreme anxiety, and self-hate. Cognitive impairment, identity confusion, and externalizing strategies were accompanied by low self-agency. Many participants performed extreme actions during this time, including treatment ward escapes and self-harm.

*I was sitting in there and everything had been taken from me and I wasn’t really motivated to come clean (…) But then (…) I think I understood how ill I was, because at that time I sort of became sober … I turned so ill in a way, I sort of did everything in order to get out, sober, to get out and get high again, I jumped out of windows landing several floors below, broke out the main door … sick things like … and the brain, well … I had no contact or control, I don’t know, it was very uncomfortable really, just to see how desperate I got, without even being high.*

Differentiating from the social context of active use was described as challenging, and in the early phase of recovery, most participants still saw drug use as part of their social identity. Many had idealized the positive effect of using drugs, and some said they enjoyed the chaos associated with this lifestyle. Moreover, drugs were described as their main strategy for coping with emotions and stressors. These perceived benefits fuelled a strong ambivalence: should they surrender and accept help, or start using drugs again?

*I like chaos. Go get some, sell some, get some more, and being in that, and maybe some war and a bit of hell, and, you know. I thought that just ruled, I found it so boring when there was no action around me.*

### Submitting to Treatment: A Struggle to Balance Rigid Treatment Structures With a Need for Autonomy

Submitting to a treatment setting and overcoming initial biological abstinence gave most participants an increase in self-agency and the belief that they might manage a drug-free life. Many highlighted that believing in change was a pre-condition for actual change and that an increase in agency thus became a catalyst for realizing positive change. However, greater agency led in most cases to a greater desire for autonomy. Over time, this required credible transcending roles (e.g. more advanced work tasks) and expectations they perceived as associated with structured treatment regimens [e.g., Narcotics Anonymous (NA)] they had initially surrendered to. They perceived this early differentiation as necessary for progression. However, many felt particularly vulnerable at this stage as it was characterized by limited life skills, drug cravings, and a fragile, only partly committed psychological structure. A common end point to this struggle was the acknowledgement that structure was needed, but only alongside individually tailored treatment.

*One is heavy therapy. The other is heavy routine (…) To process it and get through it is more important than learning to pull your pants up and get out of bed, kind of. But routines also keep things in check. So I would say it’s a good mixture.*

As the acute biological abstinence symptoms ended, most participants wanted to focus more on finding suitable meaningful activities. They often described individual or group psychotherapy as positive in this regard. However, many participants saw other, more practical treatment forms as more helpful. They described having been mentally and physically restless and tense. Also, many participants were skilled in manual labor (e.g., carpentry). They were motivated by the perceived transference value between treatment activities and potential future employment. Physical activity was also seen to alleviate feelings of emptiness and loneliness, as well as serving as a distraction from the state of abstinence. Several felt that recovery at some point had to arise from their own preferences and resources. To many participants, a lack of such relevant options from the treatment provider or social network sparked a need to differentiate.

*Yes, they had lots of you know psychologists and groups and blah blah, but I don’t feel that stuff has done much for me (…) But when I came to XX I got my hands dirty alongside that janitor guy, cutting the grass, doing some carpentry, laying some vinyl flooring and stuff, that’s dynamite for me. So that was important to me.*

### Surrendering to Trust and Love: Building a Whole Person

Coming from a social environment in which paranoia was perceived as an adaptive requirement for survival, all participants found trust difficult in the beginning. Trusting or relying on others was almost unthinkable. Establishing trusting relationships or even acknowledging a need for social dependence was highly anxiety provoking. Experience of any strong emotion, from love and joy to sadness and frustration, often triggered a desire to use drugs rather than a need for relational closeness.

*That’s what costs me more than any of it almost, out of all I have been through, it is in a way working with letting people in and caring about people, and let them care about me and … so I haven’t sort of been ready for that either, until now. And then, when I’ve been drug free for shorter periods of time I’ve not been in a position to work with it, and maybe then it loses a bit of meaning too. If you didn’t have anyone close, it might have been easier to keep using.*

Later in the course, most participants explicitly expressed that expanding their emotional tolerance window was their main tool in establishing lasting, drug-free social networks, in remaining drug free, as well as in creating a meaningful life. In order to build emotional resilience, they described having to adapt a mindset that others could be trusted, alongside exposure over time to drug-free peers and normal social settings, such as sport clubs, school, etc.

*I’ve worked very hard to keep loneliness from … just because you are alone it doesn’t mean you are lonely. I feel … I do catch myself in it sometimes that I like to be around people, and if I spend too much time alone I have to go visit someone or hang with someone, you know, but it’s good to know … or know yourself at the level that “no, you can take quite a bit before it gets bad.”*

Trust and self-esteem were seen by most participants as key to coping with a full range of emotions. Intrusive feelings of shame, self-hate, and guilt meant that achieving this required tenaciousness. Emotional coping could not be developed solely through socializing. Where drugs previously blunted feelings, current sobriety made feelings available, which also meant having to cope with previous unprocessed losses and difficulties. Participants saw basic self-acceptance as the foundation for their next steps toward recovery.

The first time I did it was completely weird. You have your own room and the door to the bathroom was closed. I remember standing there with my dentures in one hand and my toothbrush in the other, I turn around to check they can’t hear me. I squealed a few words there: “Love you, L.” I thought, “What a load of bull.” I started doing it every day and every time I passed a mirror: “I actually love you.” Then, it was also totally unbelievable, one morning I had done it so many times it started to seem normal. I felt nothing, it was just a thing I did. So when I stood there tired and awful with my dentures I said: “I love you, L.” Yes, and then I felt that I meant it. I stopped in my tracks and just “what the f*ck.” It made me a bit happy, right, but I then thought “what the h*ll is this.” I started feeling emotions. Again, and that’s some of what I’m working on now, I want to hold onto my emotions.

### A Life More Ordinary: Surrendering to Mainstream Social Responsibilities

In order to make sober life meaningful, drug-related elements of identity had to be replaced. Most participants initially felt hesitant to take on ordinary prosocial roles due to a conviction that “regular life” was boring and limited. However, alongside the integration of new roles, such as that of being an employee or drug-free friend, this conviction was gradually replaced with an appreciation of being accepted and needed. Throughout recovery, such personal affiliations gave rise to a wider sense of being a fully included member of society.

*The feeling that I am needed by others, I think that’s important. Because if I feel useless, I act useless. Working with my colleagues has made me take a role at work which makes it easier for me to stay away from drugs. My closest colleagues have meant a lot to me.*

Over time, most participants felt the need to distance themselves from initial work tasks. They came to see these as monotonous, too simple, and detrimental to growth. They felt treated as if they were second-best, which in some cases increased drug cravings. Most participants were explicit that they needed performance-based progress at work. They saw work as an arena for learning how to handle feedback and responsibility. Acceptance as an equal by work colleagues was a payoff for accepting the confines of a life more ordinary and for moving toward identification with their employee role. These processes were perceived as key to eventually seeing oneself as an on-par citizen.

*Before I got the new position I felt that “something has to happen now.” I don’t know what it will be, but I sort of need new things to learn, new challenges, to develop.*

Colleagues at work, and learning something at work, right, it was awesome to be able to tile a fat bathroom, right, or to build a chimney. I mean, I could drive around XX and point out: I worked there, I did that, a mall, you know, I tiled that sh*t. I did that, that’s quite cool.

### Accepting Personal Responsibility and Autonomy: It Has to Be Me, It Cannot Be You

For most participants, later-stage recovery was described as living life flexibly yet in line with personal values and preferences. They described a feeling of personal wholeness, based on autonomy, resilience, and integration. These self-perceptions provided support for consistent self-agency and flexibility in the face of life challenges. Relapse was, at this point, mostly perceived as a minor issue.

*It’s myself. I am the captain of my ship. It would hurt as much the next time, if a life crisis like that were to come, that someone dies or … But my experience indicates that I most likely will do the right thing then. Because I have done so earlier.*

For most participants, long-term recovery required self-acceptance and overcoming self-stigma. They had to accept themselves as reformed, drug-free persons with weaknesses and peculiarities and to internalize a feeling of being *good enough*. Combating self-stigma required letting go of negative prejudicial beliefs, such as, “Once a drug addict, always a drug addict” or “I have less value than other people.”

*I could run a marathon every day, you know … I have so much energy it almost drives me nuts. But I get some of it out with the geckos, I get quite a bit out with the kids, and then I try to get some out with friends too. So it works, in a way. I am growing chillies at home at the moment. The world’s spiciest. So something is still left there, but you just have to change certain things a little. But a guy once said to me that you should never entirely stop being a criminal. You should always hold on to the devil inside, or you’ll go to hell again quickly. It gets too boring. When you’re used to hell on wheels. So I think that’s actually a bit smart.*

The later phases of recovery investigated in this study were often associated with a sense of citizenship, stable abstinence, and high-level functioning, including competitive work and a drug-free social network. Many participants had lost interest in their former addict lifestyle, and remaining drug-free required less effort than before. Many also ended their rehabilitation community memberships (e.g., NA) during this phase. They explained that being a community member was incompatible with a fully autonomous life.

*I dunno (laughs). I don’t think about it much anymore, I just kind of get up and start the day. I don’t have many fixed routines. I am very down to earth, I just get up and drink coffee, and then I’m off really.*

*Then it was the World Championship in Big Book knowledge. I couldn’t find my place there [in the NA]. At the beginning it was OK because it was nice just having someone who knew what it was all about, but as time passed and I started thinking more on my own and my head got clearer then “no, I don’t think so.”*

## Discussion

### A Proposed Transitional Model of Surrender and Differentiation Across Resulting Themes

**Figure 1:**
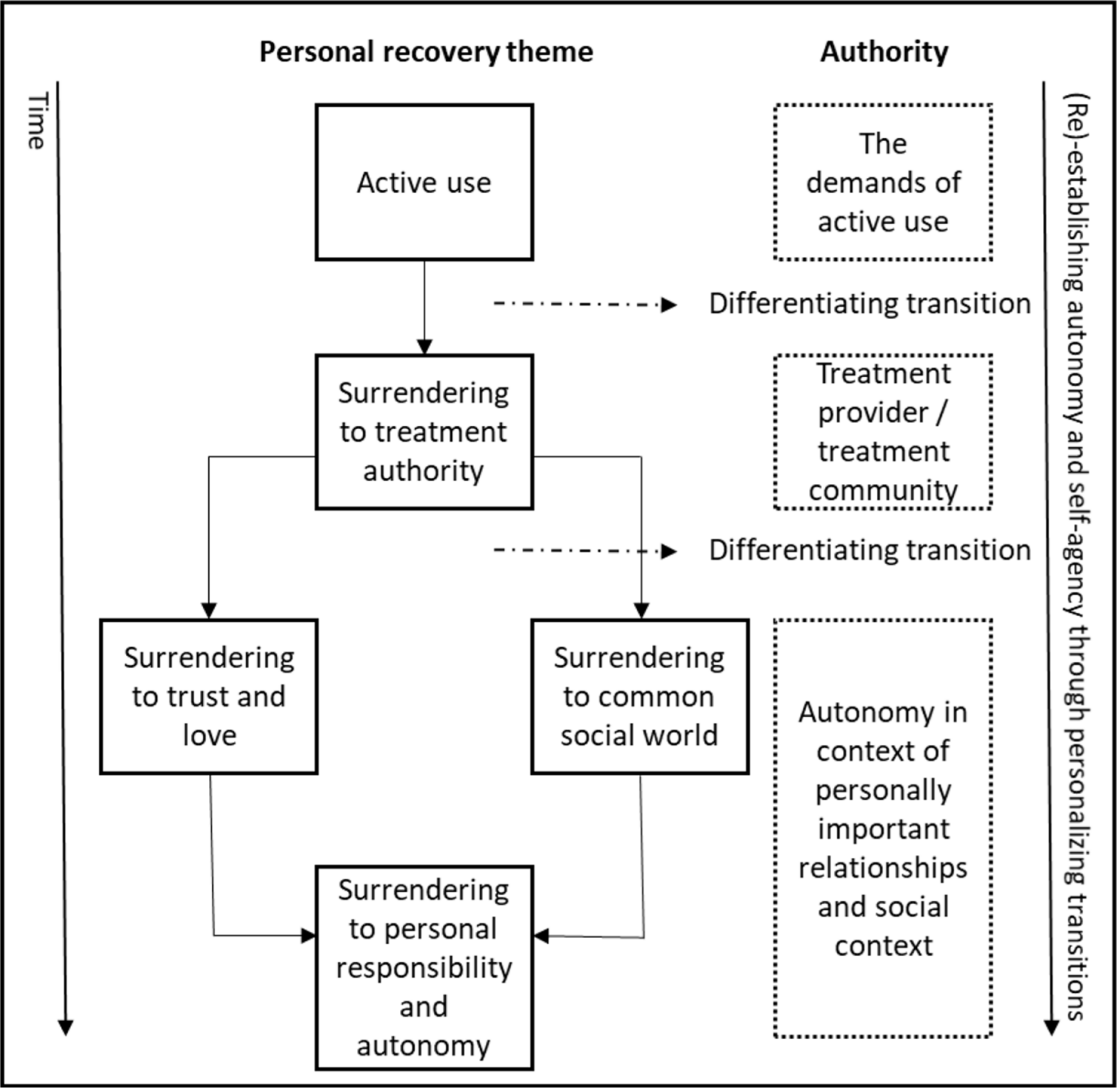
A proposed transitional model of surrender and differentiation across themes.

Reflecting previous research (3, 4, 39, 40), our analyses present long-term recovery as a challenging developmental process of moving from dependency and reactivity to personal autonomy and self-agency. Two dimensions seem particularly key in describing this process (see **Figure 1** for model).

First, *transitions*, resulting from progression and change of priorities and needs, seem to appear throughout the thematic content. Second, the recovery process entails a constant flux of surrender to authority followed by differentiating from that very authority. This rollercoaster of dependence on the one hand and independence and self-agency on the other permeates the entire recovery process and appears a motivational force moving the individual through each transition. Here, authority must be understood broadly as any external source of knowledge or instruction that is useful at one point in time, even if it is incompatible with independent thinking and actions in the long run.

The model comprises two important differentiation phases resolved by individuals as they gradually gain improved self-agency (41, 42). A proto-narrative may serve to illustrate this: during active drug use, social, biological, and psychological authoritarian forces chain participants to their status quo. The demanding differentiating from active use requires not only immense determination but also a credible alternative source of authority to tip the scales in this major decision. In our study, this new authority was usually a treatment program or a peer organization such as NA, which provided participants with evidence of attainable alternative lives. Family, friends, and peers were often important in supporting participants to submit to these alternative authorities.

Needs seemed more uniform in the early stages of recovery, with most participants describing this core transition of surrendering to new authority. In this early phase, individualism seemed sacrificed in return for the safety provided by these authorities. Later, when withdrawal symptoms abated, these same new authorities often came to be experienced as limiting, de-humanizing, or irrelevant. For many, the maintenance and progression of successful recovery thus required differentiating from the patient role or NA and the negotiation of personal freedom. A failure to differentiate was seen as posing a risk of reduced self-agency, relapse, and failed recovery.

In conclusion, recovery is described as the successful transition from immature authority support, involving problematic, but necessary, devotion to the support system’s approach, to mature authority support, involving a higher degree of independence, building on personal responsibility and individualized care. This pattern was particularly evident in the later phase of recovery, which was characterized by accounts of great variability in individual choice and values. This shift seemed to gradually build stable self-agency and constructive self-boundaries and allowed participants to grow into on-par social citizenship (1, 2). However, this general pattern does not imply that full recovery exclusively equals complete independency or that a person must differentiate himself utterly from all the more structured supports that were helpful early in recovery. Moreover, the process is seldom linear, with some individuals moving back and forth between stages. In the study sample, there was great variability, throughout the entire recovery process, to which degree participants felt a need to keep those supports to continue to stay abstinent and become productive citizens.

### Implications

Recovery was described as fragile and fluctuating, requiring combined structural change (43), such as a proper and safe living residence and successful early decision making as well as long-term commitment. Like previous results (27, 44), our findings support that service users must be involved with social networks and treatment professionals to forge functional solutions, preferably based on the person’s existing skill set and individual preferences (8). Given that exhausted relatives are the rule rather than the exception at the start of any drug abuse recovery journey, treatment professionals are crucial in presenting early functional alternatives. Participants only occasionally managed this positioning alone and described well-timed and individualized support as key to remain motivated. In particular, care that was sensitive to phase-specific needs, but also to changes in needs, seemed decisive for progression to continue. A need that was relevant early—for example, handling paranoia—was less relevant or was even perceived as counterproductive if persecuted at a later recovery stage. Echoing previous research, coherence between personal needs and the care system’s preferences and focus seemed decisive to maximizing the self-reinforcing effects of early drug freedom and emerging agency (45, 46), and for a drug-free lifestyle to be perceived as a realistic long-term alternative.

Although the actual reduction of substance use is a cornerstone of recovery, our findings also highlight social factors as imperative to quality of life (47) and long-term success (10). While some research supports this view, including how people with combined substance and psychological problems desire to fit with their peers (48), main trends in the SUD field, particularly outcome research, overlook this perspective by mainly studying individualistic models and measures that describe recovery simply in terms of personal abstinence from drug use (10, 49). The content of recovery processes changes over time as coping strategies and functioning improve (4), with treatment needs changing accordingly. Reflecting previous research, findings suggest that, for many, during the acute phase, key needs are drug reduction in a professionally provided, highly structured drug-free social setting. However, later-stage social recovery requires the individual to embark on a highly personal process of personal responsibility and real-life social adaption to a drug-free lifestyle. Reflecting previous findings (50–52) (53), these processes seem often more protracted and require continued tenaciousness and finely timed facilitation within the framework of individualized support.

In this study, time-limited, short-term treatment seems ineffective for long-term social recovery. In fact, our findings illustrate that even in a SUD sample with good prognosis, recovery is dependent on intense, long-term, and individually tailored support. Current SUD treatment structures almost unanimously fail to offer such care. Our findings highlight how current policies of early treatment termination might very well be preventing people from attaining the later stages of recovery. In this study, only at a late stage does stronger self-agency allow recovering drug addicts to maintain recovery independently. As a consequence, our findings highlight a considerable problem for common clinical practice, where early termination and lack of individual tailoring and process awareness might sabotage any early recovery successes.

### Limitations

The main limitation concerns representativeness of the sample. This is a clinical sample recruited in the beginning of a new treatment episode. We do not know if the same findings would be obtained in people who recover without formal treatment. A high percentage of participants had good functioning levels prior to SUD. Hence, this was a relatively homogeneous group of good-prognosis patients, as would be expected when using social recovery as an inclusion criterion. However, this is not to say that these patients were not at risk of long-term functional disability. Also, this does not compromise the validity of the findings, even if it limits generalizability to the most severe and prolonged SUD conditions. A second limitation concerns contamination. This study does not perform the isolation of single factors aiding recovery, e.g., that of therapy versus that of medication or social support.

## Ethics Statement

This study was reviewed and approved by the Regional Ethical Committee (2011/1877-REK Vest) and conducted according to its guidelines and those of the Helsinki Declaration (1975).

Participants gave their informed written consent.

## Author Contributions

All authors have made substantial contributions to all phases of the paper. JB, MV, CM, TSS, AE, JM, and SN contributed to concept development and interviews, performed analyses, and wrote the first draft. TES and AS performed the interviews and contributed in analysis. All authors were involved in study design, provided scientific oversight throughout the project, detailed comments on the paper across several drafts, and edited the paper.

## Funding

This work was supported by internal financing from the Centre for Alcohol and Drug Research at the Stavanger University Hospital. The funding source provided no input into the analyses or presentation of these data.

## Conflict of Interest Statement

The authors declare that the research was conducted in the absence of any commercial or financial relationships that could be construed as a potential conflict of interest.
